# Nuclear magnetic resonance/single molecule fluorescence combinations to study dynamic protein systems

**DOI:** 10.1016/j.sbi.2023.102659

**Published:** 2023-10

**Authors:** Ida Marie Vedel, Andromachi Papagiannoula, Samuel Naudi-Fabra, Sigrid Milles

**Affiliations:** Leibniz-Forschungsinstitut für Molekulare Pharmakologie (FMP), Robert-Rössle-Str. 10, 13125 Berlin, Germany

**Keywords:** Single molecule FRET, NMR spectroscopy, Protein dynamics, Intrinsically disordered proteins

## Abstract

Many proteins require different structural states or conformations for function, and intrinsically disordered proteins, i.e. proteins without stable three-dimensional structure, are certainly an extreme. Single molecule fluorescence and nuclear magnetic resonance (NMR) spectroscopy are both exceptionally well suited to decipher and describe these states and their interconversion. Different time scales, from picoseconds to several milliseconds, can be addressed by both techniques. The length scales probed and the sample requirements (e.g. concentration, molecular weight, sample complexity) are, however, vastly different, making NMR and single molecule fluorescence an excellent combination for integrated studies. Here, we review recently undertaken approaches for the combined use of NMR and single molecule fluorescence to study protein dynamics.

## Highlights

• NMR and single molecule FRET (smFRET) cover highly complementary distance ranges.
• Both techniques are sensitive to protein dynamics across various time scales.
• Conformational exchange of proteins have been studied by integrated NMR and smFRET.
• Integration of NMR and fluorescence allow more robust models for protein dynamics.

## Graphical abstract

**Figure:**
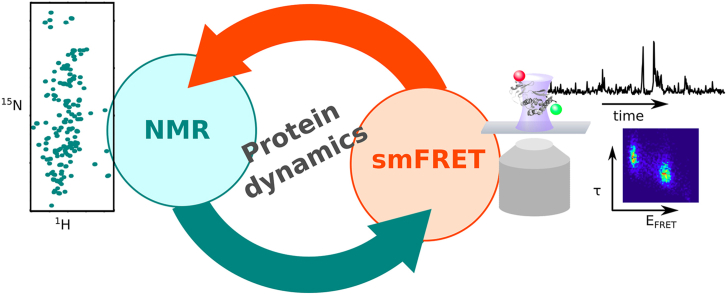

## Introduction

**Figure 1:**
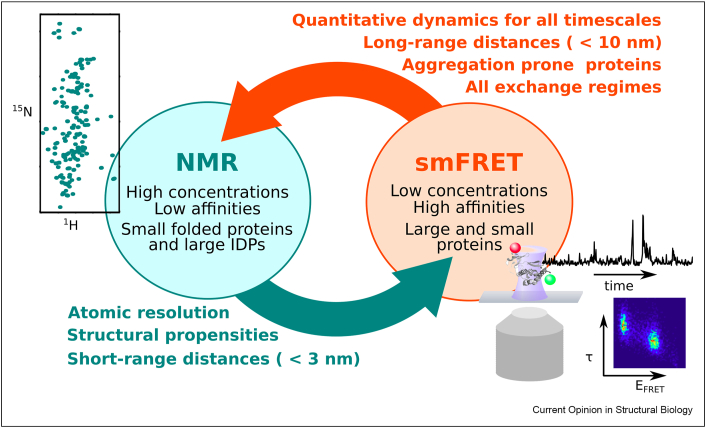
**Synergies between NMR and smFRET.** Sample requirements of the two techniques are in black in the turquoise (NMR) and orange (smFRET) circles. Strengths and specific information available from the two techniques are written in turquoise and orange, respectively. A ^1^H–^15^N HSQC spectrum of an IDP is shown on the left. On the right is an illustration of a confocal detection volume with a double labeled protein, a single molecule time trace (black), and a fluorescence lifetime (τ) versus FRET efficiency (E_FRET_) histogram.

Elucidation of proteins’ three-dimensional structure is without a doubt crucial to understanding their working mechanism. While some proteins function by the mere presence of their structure as static components of the cell, most proteins have to undergo conformational changes to fulfill their tasks. Nuclear magnetic resonance (NMR) and single molecule fluorescence spectroscopy, both solution state techniques, are extremely well suited to investigate such dynamic movements, and even permit the analysis of proteins without stable three-dimensional structure, called intrinsically disordered proteins (IDPs) [1, 2, 3]. NMR generally provides molecular insight into dynamic protein systems and offers a plethora of different parameters to assess protein motion. Accessible length scales are, however, limited to around 2.5 nm at maximum through paramagnetic relaxation enhancements (PREs) [4], whereby the distances between a site-specifically attached spin radical and all backbone N–H bonds are measured. Single molecule fluorescence, in particular single molecule Förster resonance energy transfer (smFRET), provides access to longer length-scales - up to 10 nm - by measuring the distance between a site-specifically attached donor and acceptor fluorophore [1,5]. NMR and smFRET also complement each other efficiently on other aspects (Figure 1): Large protein size or conformational exchange in the microsecond to millisecond range, for example, often lead to severe NMR line broadening. While NMR exchange techniques can be used to exploit this phenomenon [6,7], extreme line broadening can prevent NMR analysis altogether. Even though smFRET relies on the attachment of fluorophores and can thus only measure distinct distances, fluorescence techniques do not suffer from the same drawback and can be used to analyze dynamics at various time scales even for large protein systems.

**Figure 2:**
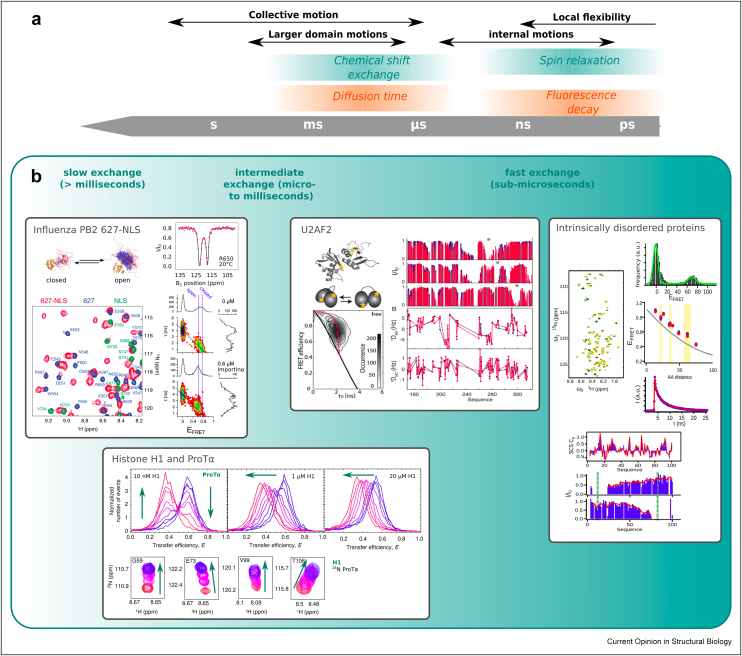
**Dynamic timescales in NMR and smFRET. (a) Time scales of relevance for biological systems.** Slow exchange behaviour is generally characterized by the observation of two (or more) distinctly appearing states, e.g. in the NMR spectrum or in a FRET histogram. Fast exchange is the other extreme, by which the characteristics of the states in fast exchange are completely averaged as a function of their respective populations. Intermediate exchange can sometimes be harder to interpret and can result, for example, in line broadening in NMR spectra or broadening of FRET peaks in the histogram. Central parameters with respect to which dynamic exchange is relevant are fluorescence lifetimes and NMR spin relaxation as well as the diffusion time and the chemical shift difference between two states, respectively, covering similar time scales. NMR parameters are in turquoise, smFRET parameters in orange. Adapted from Ref. [8]. **(b) Examples for NMR/smFRET combinations for different exchange regimes. Influenza PB2 627-NLS:** Scheme of open and closed conformational ensemble. ^1^H–^15^N correlation spectrum of 627-NLS (red), and the 627 (blue) and NLS (green) domains alone, demonstrating the presence of an ‘open’ and a ‘closed’ state. CEST profile of an exemplary residue in slow exchange. τ versus FRET efficiency (E_FRET_) histograms of 627-NLS labeled with one fluorophore on the 627 domain, the other on the NLS domain, showing the 0-FRET peak, where the acceptor dye is absent, and a FRET peak encompassing both open and closed states in slow exchange. In the presence of Importinα, the closed state disappears from the histogram. Adapted with permission from Ref. [15]. Copyright 2015 American Chemical Society. **Histone H1 and ProTα:** FRET histograms of doubly labelled ProTα in the presence of 20 μM, 1 μM and 10 nM H1 supplemented with no or increasing amounts of unlabelled ProTα, resulting in fast, intermediate and slow exchange respectively. Below are zooms into ^1^H–^15^N HSQC spectra of ^15^N labelled ProTα showing peak movements upon addition of H1 in agreement with 4-state kinetics allowing for 1:1, 1:2 and 2:1 binding ratios between the two partners. Adapted from reference [26∗∗]. **U2AF2:** Structure of RRM1 and RRM2 domains in their closed conformation and scheme of open and closed conformations. Positions of the FRET fluorophores are indicated by stars. Below: FRET versus fluorescence lifetime (τ) histogram showing deviation from the static FRET line (black) and description of the two state dynamics (red line). Adapted from Ref. [18]. Right: Three sets of PREs and 2 sets of RDCs (blue: experimental, red: calculated from a conformational ensemble). Adapted with permission from Ref. [17]. Copyright 2014 American Chemical Society. **Intrinsically disordered proteins:** Shown are NMR and smFRET data acquired on the IDP P_1-100_. A ^1^H–^15^N HSQC spectrum in the absence (green) and presence of a PRE label, a FRET histogram (black) with fit (green), FRET efficiencies plotted against the amino acid distance between the labels, a fluorescence lifetime decay, secondary C_α_ chemical shifts (SCS C_α)_, two sets of PREs plotted against the amino acid sequence (blue: experimental, red: calculated from a conformational ensemble, above yellow background are cross-validated data). Adapted from Ref. [43∗∗].

In this review, we shed light on recent advances undertaken to use both NMR and single molecule fluorescence for the analysis of protein dynamics in various biological systems. We point out how the two techniques complement each other in the different studies (Figure 2) and discuss the potential of NMR and single molecule fluorescence combinations for future applications to study protein dynamics.

## Different exchange regimes studied by NMR and smFRET

When studying protein dynamics by NMR or smFRET, the time scales on which these dynamics occur are crucial as they determine how different experimental parameters are averaged. Those can be spin relaxation or the chemical shift in NMR, and the fluorescence lifetime decay or the diffusion time for smFRET based on a confocal detection geometry (Figure 2a) [8]. While, strictly speaking, an exchange regime has to be defined contextualized within the scope of each specific experiment, we categorize these regimes or time scales as follows: slow (milliseconds to seconds), intermediate (microseconds to milliseconds) and fast (few nanoseconds and sub-microseconds) exchange. This serves as a good approximation for both chemical shift information and the protein diffusion time in the confocal volume. Note that, even though smFRET can be observed using confocal detection on diffusing (or immobilized) molecules, or using total internal reflection illumination (TIR) on immobilized molecules [5], combinations of NMR and smFRET have in the past focused on experiments using confocal fluorescence setups. In this review we thus concentrate on experiments acquired on confocal setups, which in contrast to TIR illumination, permits access to dynamics on the microsecond timescale or faster.

### Slow exchange

When the dynamics of a system, e.g. the interconversion between two different protein conformations, occur on a slow time scale, the experimental parameters of both states can be distinctively and simultaneously observed.

Slow exchange dynamics was observed in a study of the trans-activation response RNA binding protein (TRBP), which is implicated in the RNA interference (RNAi) pathway and consists of three dsRNA binding domains. A construct comprising two of these domains, linked by an intrinsically disordered linker, has been described to bind RNA in two major binding poses using intermolecular nuclear Overhauser effects (NOEs), sensitive to short distances of only a few Å between two NMR active nuclei (see also Box 1 for a brief explanation of the different NMR and fluorescence techniques mentioned in this review), and electron paramagnetic resonance (EPR), which probes distances up to around 6 nm between two site-specifically attached spin labels (DEER, double electron–electron resonance [9]). Two binding poses were proposed as no single pose could satisfy all the measured distances derived from NOEs and PREs. Residual dipolar couplings (RDCs), sensitive to angular averaging, revealed that the orientation of the two domains with respect to each other was identical for both poses. Two different labeling positions for donor and acceptor dyes for FRET were strategically designed with one label on the protein and one on the RNA, allowing distinct FRET efficiencies for the two states in slow exchange, rather than a single FRET efficiency, permitting to quantify their ratio and thus resolving the remaining structural ambiguity [9].

Box 1 **Description of NMR and single molecule fluorescence experiments mentioned in this review.**

**NMR experiments**

**Chemical shifts** are particularly sensitive to the local environment (especially ^15^N and ^1^H) and to secondary structure (^13^C backbone chemical shifts). They describe where in the spectrum an NMR peak is located [58].

**NOE:** Transfer of nuclear spin polarization from one nucleus to another one close in space with a distance dependance of 1/r^6^. Distance restraints derived from NOE data are usually smaller than around 6 Å [59].

**J-couplings**, also called **scalar couplings** are through-bond couplings and depend on dihedral angles. They are thus sensitive to protein structure [60].

**Residual dipolar couplings (RDCs)** probe the angular averaging between a chemical bond and the magnetic field. They are used to determine the orientation of molecular domains [61].

**Chemical exchange saturation transfer (CEST):** The effect of a weak saturating radiofrequency field applied at different frequencies on an NMR peak (peak 1) is detected. An intensity drop is observed when the field is at the frequency of peak 1 or another peak in exchange with peak 1 (peak 2, visible or invisible). The chemical shift difference between the two states (corresponding to peak 1 and peak 2), the exchange rate, as well as the relaxation rate and population of state 2 can be detected [11].

**Spin relaxation** (e.g. R_1_, R_2_, heteronuclear NOE) are characterized by protein motion in the picosecond to nanosecond range [62]. Transverse relaxation (R_2_) is sensitive to intermediate exchange in the microsecond to millisecond regime [63].

**PRE:** Spin labels (usually nitroxide radicals) introduced into proteins cause enhanced relaxation of nuclei in their spatial proximity. Distance information up to around ∼30 Å can be obtained [4].

**Fluorescence experiments**

**Single molecule Förster resonance energy transfer (smFRET) histograms** are obtained by calculating the efficiency of energy transfer between two fluorophores site-specifically attached to a protein. This is usually done with the help of per-molecule donor and acceptor fluorescence intensities. They provide information on distance (and their distribution) between the fluorophores (up to ∼10 nm) [5].

**Fluorescence lifetime** is the average time a fluorophore spends in the electronically excited state before returning to the ground state. The lifetime of a FRET donor fluorophore is shortened by the proximity of an acceptor to which energy transfer occurs [5].

**Fluorescence anisotropy** refers to the (average) polarization of fluorescence emission. Anisotropy is influenced by the degree of rotational motion, and therefore on the size and shape of the fluorescent molecule [64].

**(Nanosecond) Fluorescence Correlation Spectroscopy (FCS/nsFCS)** analyses the time-dependent fluctuations of fluorescence intensities within the confocal volume. Fitting these data to a model allows characterization of diffusion times, concentration and size of the protein [64]. In the fast time scales (nanoseconds), the method is sensitive to protein dynamics of a FRET labelled sample [22].

**Photon Distribution Analysis (PDA)** is a statistical method that is used to analyse the width of FRET efficiency distributions as a function of the number of states and dynamic exchange on the microsecond to millisecond time scale [20,65].

Alt-text: Box 1

The influenza polymerase protein PB2 also shows hallmarks of slow exchange conformational dynamics within its two terminal domains 627 and NLS. In its ^1^H–^15^N correlation spectra, the two domain protein construct 627-NLS showed nearly twice as many peaks as expected based on its protein sequence, arguing for the existence of two (rather than one) states in slow exchange with respect to the chemical shift difference between the two states. Those states have been interpreted as both a closed state, also described by its crystal structure [10], and an open state, in which the two domains rotate freely with respect to each other. This conclusion was based on a perfect spectral overlap of the individual 627 and NLS domains with one set of the peaks in the 627-NLS spectrum, showing that the chemical shifts of the individual domains were not affected by the chemical environment of the respective other domain. The exchange rate between open and closed states was determined by chemical exchange saturation transfer (CEST [11], see also Box 1) to be 50 s^−1^ at 25 °C with an occupancy ratio of 1:1 (open:closed). This measured interconversion timescale resulted in two separate FRET populations for the open and closed states, since the interconversion rate was significantly slower than the protein diffusion time through the observation volume. Additionally, the relationship between FRET efficiency and fluorescence lifetime [12] of the open state agrees with a highly dynamic ensemble in fast exchange as also observed by NMR. Fluorescence lifetimes are sensitive to much shorter time scales than intensity based FRET efficiencies and consist of a multi-exponential decay if dynamics slower than a few nanoseconds exist. While slow exchange processes, such as the exchange between the open and the closed state, result in two well separated FRET (and fluorescence lifetime) populations, exchange faster than the inter-photon time (usually in the microsecond range), leads to an averaging into one FRET population with, however, multi-exponential lifetime decay resulting in a longer average fluorescence lifetime as compared to a static FRET population [12,13]. Thanks to its insensitivity with respect to protein size, smFRET was furthermore able to show binding of 627-NLS to the nuclear transport receptor Importinα through the NLS domain (named after its nuclear localization sequence [14]), and revealed that only the open conformation was binding competent (Figure 2) – a result also in agreement with small angle X-ray scattering (SAXS) [15].

### Fast/intermediate exchange

In the case of fast exchange dynamics, the experimental parameters of the different states are averaged depending on the abundance of each state and observed as one experimental parameter. All experimental parameters can only be satisfied if a multi-state model is evoked, whereby the parameters corresponding to the different states are averaged. A multi-technique study using SAXS, PREs and RDCs investigated the domain dynamics of two RRM(RNA recognition motif)-domains of the splicing factor U2AF2 connected via a disordered linker, for which an open and a closed state in equilibrium had been previously identified [16]. While PREs were consistent with compact conformations, more extended conformations were required to describe the SAXS data, which was satisfied by a pool of ‘detached’ conformations, in which both domains sample various distances and orientations with the help of the disordered linker connecting them (addressed using RDCs) [17]. All different conformations are sampled in fast exchange, in agreement with all NMR parameters and the relationship between fluorescence lifetimes and intensity based FRET efficiencies (see also previous paragraph) of a sample containing a donor and an acceptor fluorophore on the two domains, respectively [18]. More recently, a multi-laboratory study has revealed the presence of ‘intermediate exchange’ leading to broadening of the intensity-based FRET peak [19], which was analyzed using photon distribution analysis (PDA) [20]. Fluorescence lifetimes, which are subject to different averaging and short compared to the observed dynamics, have thus been used to assess the previously built ensemble using a multi-exponential lifetime decay [19]. This example demonstrates the importance of considering the time scales a certain parameter reports on and illustrates the difficulty of classification into different exchange regimes.

### Protein systems sampling different dynamic timescales

While some of the above examples, particularly 627-NLS from the influenza polymerase protein PB2, already showed exchange dynamics on different time scales, the following example demonstrates a shift in exchange dynamics as a function of protein concentration: Histone H1, a linker histone transiently associated with the nucleosome dyad and involved in chromosome condensation [21], is comprised of a folded core domain, a short N-terminal and a long C-terminal intrinsically disordered region. Its chaperone, prothymosin α (ProTα), is purely disordered and of opposite (negative) charge, and both smFRET and NMR experiments have demonstrated the interaction between these two proteins. Doubly FRET labeled ProTα showed the presence of one FRET peak characterized by very rapid dynamics between the two fluorophores as demonstrated by nanosecond FCS, which probes the different dynamic timescales directly through correlating the fluorescence intensities of the FRET donor and acceptor (or cross-correlating donor and acceptor fluorescence) upon excitation of the donor fluorophore [22]. Upon addition of picomolar amounts of H1 to fluorescently labeled ProTα, the number of molecules with a FRET efficiency reminiscent of the unbound state decreased and a new distinct FRET efficiency, interpreted as a bound state, appeared – a classical slow exchange signature. The resulting extremely high (picomolar) affinity between H1 and ProTα was confirmed by intermolecular FRET experiments. NMR titrations revealed only small and gradual chemical shift changes, suggesting that the proteins maintained their overall disordered state upon interaction with a signature of fast or intermediate exchange [23]. The molecular binding rates governing this process have been addressed in a follow-up study, where unlabeled ProTα was titrated into FRET-labeled ProTα in the presence of H1. Depending on the initial amount of H1 present, slow, intermediate or fast exchange behavior was observed leading to either two distinct bound and unbound FRET populations or gradually shifting peaks broadening at intermediate exchange with respect to the diffusion time (Figure 2b). SmFRET of immobilized ProTα that remains in the observation volume of a confocal microscope for a longer time, recurrence analysis (enabling the observation of slower dynamics due to molecules diffusing back into the confocal volume [24]) and fluorescence stopped flow experiments were used to quantify binding rates between the two proteins and revealed that, at higher protein concentrations, higher binding stoichiometries were obtained. Calculations of ^15^N lineshapes based on these kinetics [25] revealed that those higher stoichiometries also explained the experimental NMR spectra of ProTα interacting with H1 [26∗∗]. Not only does this example show that different concentrations of protein can lead to very different behavior of a protein system leading to its classification into different exchange regimes, but it also demonstrates the added value for assessing protein behavior at different concentrations, which smFRET can offer through the addition of unlabeled protein in the sample.

### Quantitative integration of smFRET and NMR for conformational ensembles of IDPs

IDPs have classically been associated with fast exchange dynamics within their protein chains observed by both NMR and smFRET spectroscopy [23,27]. These dynamics have led to the description of IDPs as statistical chains to interpret smFRET data of doubly labeled proteins [28, 29, 30]. Concomitant NMR and SAXS studies have described IDPs as conformational ensembles, groups of protein conformations, that together describe the experimental data (e.g. chemical shifts, RDCs, PREs) [31, 32, 33]. The fruitful combination of smFRET and NMR for the study of IDPs and their complementarity in terms of distances probed, has encouraged the development of integrated strategies to calculate conformational ensembles for the description of IDPs, for which various strategies have been exploited. IDPs show exchange dynamics on such rapid time scales, that fast exchange characteristics usually persist with respect to all NMR parameters assessed in the studies below, as well as for FRET efficiencies calculated from fluorescence intensities of the donor and the acceptor. Fluorescence lifetimes are short compared to IDP dynamics, which is why multi-exponential decays are present and need to be considered.

In a study of urea unfolded ubiquitin, ensembles comprised of 10 conformers were calculated using restrained structure calculations performed with XPLOR-NIH [34] and its ensemble calculation module [35]. For this, a set of extended starting structures has been individually randomized by torsion angle dynamics and subsequently matched the experimental RDCs and PREs through a simulated annealing protocol [35]. J-couplings and SAXS were included later on [29]. Intensity-based FRET efficiencies calculated from the number of emitted donor and acceptor photons as well as fluorescence lifetimes were calculated from smFRET experiments using 7 different labeling positions and encompassing between 26 and 68 amino acids between the labels. Distance distributions between the labels were calculated based on a Gaussian chain model assuming a contribution of the dyes and their linkers equivalent to the length contributed by 9 additional amino acids as determined through a global fit of all smFRET data. Ensembles derived from NMR and SAXS described the measured FRET efficiencies and fluorescence lifetimes, demonstrating very good agreement between the three kinds of data [29].

A conformational ensemble of the yeast IDP Sic1 was calculated using the statistical coil generator TraDES [36,37], from which smaller ensembles were selected using the algorithm ENSEMBLE, which matches a set of conformers to various experimental (usually NMR or SAXS) data using a switching Monte Carlo algorithm [33]. Previously recorded NMR chemical shifts and PREs [38,39] together with SAXS, were used as an input. Agreement of this ensemble with an smFRET distance was determined using the calculation of dye accessible volumes (AVs) [40]. Monte Carlo simulations of the photon emission process and Brownian dynamics simulations of the dyes let the authors conclude on dye dynamics significantly longer than the fluorescence lifetime of the donor, which was used to determine the correct time-averaging of the AV simulations. When long-range information through SAXS and PREs was used as an input, the ensemble was in good agreement with the smFRET distance [41∗].

**Figure 3:**
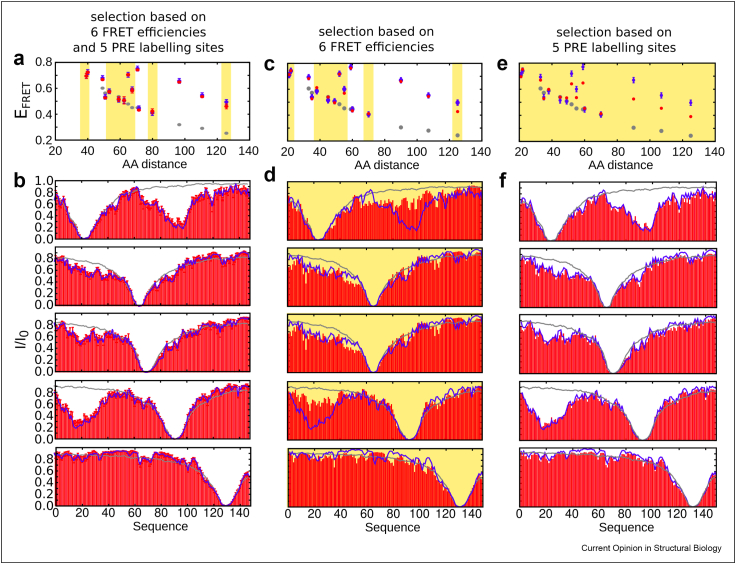
**Complementary length scales by PREs and FRET.** FRET efficiencies (E_FRET_) and PREs described by a conformational ensemble using flexible meccano [42] and ASTEROIDS [32] on *in silico* data, illustrating the different length scales the two parameters are sensitive to. 6 FRET efficiencies (E_FRET_) and 5 sets of PREs, only 6 FRET efficiencies, or only 5 sets of PREs were used as an input for ensemble generation respectively. (A,C,E) E_FRET_ plotted against amino acid distance between the attached labels for the different ensembles. (B,D,F) PREs for the different ensembles. Grey: Values expected for a statistical coil ensemble (flexible-meccano); blue: values calculated from the *in silico* data. Error bars are 0.02 and depict the allowed error in the ASTEROIDS selection. Red: Values calculated from the selected ensemble. Above yellow background are the data, which were not used in generating the ensemble (cross-validation). Adapted from Ref. [43∗∗].

A systematic *in silico* approach using the statistical coil generator flexible-meccano [42] combined with the ensemble selection algorithm ASTEROIDS, which is based on a genetic algorithm [32], has shed particular light on the complementarity between PREs and smFRET (Figure 3) and has allowed determining the number and lengths of FRET distances necessary to comprehensively probe long-range contacts in the conformational ensemble of an IDP. In this study, chemical shifts, PREs and FRET efficiencies were used to calculate the conformational ensemble, while a set of FRET efficiencies and all fluorescence lifetimes were kept aside for subsequent cross validation. The calculation of AVs was optimized for reliability and computational speed allowing the calculation of AVs across the pool of conformers (10 0000) from which the ensembles were selected. The procedure was validated on experimental PREs, chemical shifts, SAXS, FRET efficiencies and fluorescence lifetimes of the first 100 amino acids from the measles virus phosphoprotein (Figure 2) [43∗∗].

Another approach has been undertaken to characterize a construct comprising four pseudorepeat domains of tau protein. An ensemble was generated using a ‘re-weighted hierarchical chain growth’ algorithm based on a library of short fragments derived from molecular dynamics simulations. The simulated fragments were biased with the help of experimental C_α_ chemical shifts using Bayesian interference [44] and steric clashes were excluded during chain growth. A final weighing step was introduced to remove the initial bias and the resulting ensemble was compared with experimental J-couplings, RDCs and PREs. Agreement with an experimental FRET distance was tested using a self avoiding walk model assuming a contribution of the linker and dye equivalent to 4.5 residues, in agreement with previous studies [29,45], as well as by explicitly modeling atomic descriptions [46] of the dye. Minor reweighing or a small adaptation of the force field were sufficient to yield agreement between smFRET and simulations [47∗].

These quite different approaches for combining NMR and smFRET data quantitatively to characterize rapidly inter-converting ensembles of IDPs, have emerged very recently and illustrate the importance and timeliness of this research, which will certainly be expressed in future applications.

## Towards a quantitative integration of protein dynamics

Along with the development of quantitative approaches for combining NMR and smFRET into conformational ensembles of IDPs, descriptions of protein dynamics with both techniques have also been developed.

One way of going about this challenging endeavor has been through molecular dynamics (MD) simulations, from which different dynamic time scales can be calculated. Trajectories of 16 μs using the Amber12 force field with a TIP4P-D water model [48] have shown remarkable agreement with time scales of α-synuclein dynamics inferred by ^1^H relaxometry [49] (few nanoseconds) and by nanosecond-FCS [22] (tens to hundreds nanoseconds). Comparison with ^15^N spin relaxation (R_1_, R_2_, heteronuclear NOE), sensitive from picoseconds to several nanoseconds, revealed a systematic overestimation of the rates by the MD simulations, which was resolved by scaling the MD-derived order parameters corresponding to the time scales addressed by the spin relaxation experiments [50].

Order parameters (S^2^), essentially describing the relative contributions of different time scales to one correlation function, either accessed by time resolved fluorescence anisotropy and polarization sensitive nanosecond-FCS of singly labeled protein species, or by ^15^N spin relaxation, have been proposed as a common ground for resolving the picosecond to nanosecond dynamics within the folded protein GABARAP [51∗]. The study made use of the theoretical considerations presented by Lipari and Szabo [52] and reveals consistently high (around 0.9) S^2^ along the protein chain based on a ‘model-free’ analysis of ^15^N spin relaxation [53] and considerably smaller, labeling-site dependent S^2^ based on a fit of the fluorescence anisotropy decays. The NMR derived S^2^, reporting on protein backbone motion, agrees remarkably well with those calculated from MD simulations. Fluorescence anisotropy derived S^2,^ sensitive to side chain and dye dynamics, show similar trends as those calculated from the respective side chain motions in the MD simulation. However, overall protein rotational motion, to which both NMR and fluorescence are sensitive, diverge by about 19%. This divergence has been attributed to a dimerized state, which is not visible in the NMR experiments due to its large size, and thus only impacts the fluorescence-derived rotational motion [51∗].

## Conclusion

With the success of NMR/single molecule fluorescence combinations to study protein structure/conformation and dynamics, the two techniques have proven to be an excellent pair for the investigation of various dynamic time scales. In addition to the examples presented in this review, smFRET has frequently been used to support previous NMR experiments for example in aggregating protein systems that required too low sample concentrations for NMR [54], or in systems that lacked NMR peaks or peak assignment (for example due to intermediate exchange dynamics present in the sample)[55, 56, 57]. This order of experiments seems reasonable given the higher throughput and molecular resolution of NMR as compared to smFRET, where only a single distance/dynamics can be measured at the time. Nevertheless, literature has shown that smFRET has frequently provided essential knowledge to understand a dynamic protein system in combination with NMR.

In the mean time, the developments have clearly gone towards a more quantitative integration of fluorescence and NMR parameters. While this exciting development is ongoing, NMR and smFRET will certainly continue delivering answers to complex biological questions, supplementing each other when atomic resolution is needed, if the proteins are too large for NMR or aggregation prone, or if intermediate exchange dynamics complicates data interpretation.

## Declaration of competing interest

The authors declare that they have no known competing financial interests or personal relationships that could have appeared to influence the work reported in this paper.

## Data Availability

No data was used for the research described in the article.
